# Challenges to curing primary brain tumours

**DOI:** 10.1038/s41571-019-0177-5

**Published:** 2019-02-07

**Authors:** Kenneth Aldape, Kevin M. Brindle, Louis Chesler, Rajesh Chopra, Amar Gajjar, Mark R. Gilbert, Nicholas Gottardo, David H. Gutmann, Darren Hargrave, Eric C. Holland, David T. W. Jones, Johanna A. Joyce, Pamela Kearns, Mark W. Kieran, Ingo K. Mellinghoff, Melinda Merchant, Stefan M. Pfister, Steven M. Pollard, Vijay Ramaswamy, Jeremy N. Rich, Giles W. Robinson, David H. Rowitch, John H. Sampson, Michael D. Taylor, Paul Workman, Richard J. Gilbertson

**Affiliations:** 1 Department of Pathology, University Health Network, Toronto, Ontario Canada; 2 CRUK Cambridge Institute, Li Ka Shing Centre, Cambridge, UK; 3 The Institute of Cancer Research, London, UK; 4 Department of Oncology, St Jude Children’s Research Hospital, Memphis, TN USA; 5 Center for Cancer Research, National Cancer Institute, Bethesda, MD USA; 6 Telethon Kids Institute, Subiaco, WA Australia; 7 Department of Neurology, Washington University School of Medicine, St Louis, MO USA; 8 Great Ormond Street Hospital for Children, London, UK; 9 Human Biology Division, Fred Hutchinson Cancer Research Center, Seattle, WA USA; 10 Pediatric Glioma Research Group, Hopp Children’s Cancer Center at the NCT Heidelberg, Heidelberg, Germany; 11 Ludwig Institute for Cancer Research, University of Lausanne, Lausanne, Switzerland; 12 Cancer Research UK Clinical Trials Unit, Institute of Cancer and Genomic Sciences, University of Birmingham, Birmingham, UK; 13 Dana–Farber/Boston Children’s Cancer and Blood Disorders Center and Harvard Medical School, Boston, MA USA; 14 Human Oncology and Pathogenesis Program and Department of Neurology, Memorial Sloan Kettering Cancer Center, New York, NY USA; 15 Oncology, AstraZeneca IMED Biotech Unit, Boston, MA USA; 16 Division of Pediatric Oncology, Hopp Children’s Cancer Center at the NCT Heidelberg, Heidelberg, Germany; 17 Cancer Research UK Edinburgh Centre and Medical Research Council Centre for Regenerative Medicine, University of Edinburgh, Edinburgh, UK; 18 Department of Paediatrics, The Hospital for Sick Children, Toronto, Ontario Canada; 19 Division of Regenerative Medicine, Department of Medicine, University of California, San Diego, CA USA; 20 Department of Paediatrics, University of Cambridge and Wellcome Trust-MRC Stem Cell Institute, Cambridge, UK; 21 The Preston Robert Tisch Brain Tumor Center, Duke Cancer Center, Durham, NC USA; 22 The Arthur and Sonia Labatt Brain Tumour Research Centre and Division of Neurosurgery, The Hospital for Sick Children, Toronto, Ontario Canada; 23 CRUK Cambridge Institute and Department of Oncology, University of Cambridge, Hutchison/MRC Research Centre, Cambridge Biomedical Campus, Cambridge, UK

**Keywords:** CNS cancer, Cancer models, Cancer therapy, Translational research

## Abstract

Despite decades of research, brain tumours remain among the deadliest of all forms of cancer. The ability of these tumours to resist almost all conventional and novel treatments relates, in part, to the unique cell-intrinsic and microenvironmental properties of neural tissues. In an attempt to encourage progress in our understanding and ability to successfully treat patients with brain tumours, Cancer Research UK convened an international panel of clinicians and laboratory-based scientists to identify challenges that must be overcome if we are to cure all patients with a brain tumour. The seven key challenges summarized in this Position Paper are intended to serve as foci for future research and investment.

## Abstract (web summary)

Brain cancer encompasses a diverse range of complex malignancies, many of which are associated with a poor prognosis and require more effective treatments. In this Position Paper, an international panel of clinicians and laboratory-based scientists convened by Cancer Research UK identify and discuss seven challenges that must be overcome if we are to cure all patients with a brain tumour.

## Introduction

Brain tumours are among the most feared of all forms of cancer. More than two-thirds of adults diagnosed with glioblastoma — the most aggressive type of brain cancer — will die within 2 years of diagnosis^1,2^. Brain cancers are also the most common and most lethal of all paediatric solid tumours^3^. Furthermore, children with these tumours who survive and enter adulthood will often be affected by the long-term consequences of exposing the developing brain to medical interventions, including surgery, radiotherapy and/or chemotherapy^4,5^.

Brain tumours have proved challenging to treat, largely owing to the biological characteristics of these cancers, which often conspire to limit progress. First, by infiltrating one of the body’s most crucial organs, these tumours are often located beyond the reach of even the most skilled neurosurgeon. These tumours are also located behind the blood–brain barrier (BBB) — a system of tight junctions and transport proteins that protect delicate neural tissues from exposure to factors in the general circulation, thus also impeding exposure to systemic chemotherapy^6,7^. Furthermore, the unique developmental, genetic, epigenetic and microenvironmental features of the brain frequently render these cancers resistant to conventional and novel treatments alike^8–10^. These challenges are compounded by the rarity of brain tumours relative to many other forms of cancer, which limits the level of funding and interest from the pharmaceutical industry and attracts a relatively small and fragmented research community.

**Fig. 1:**
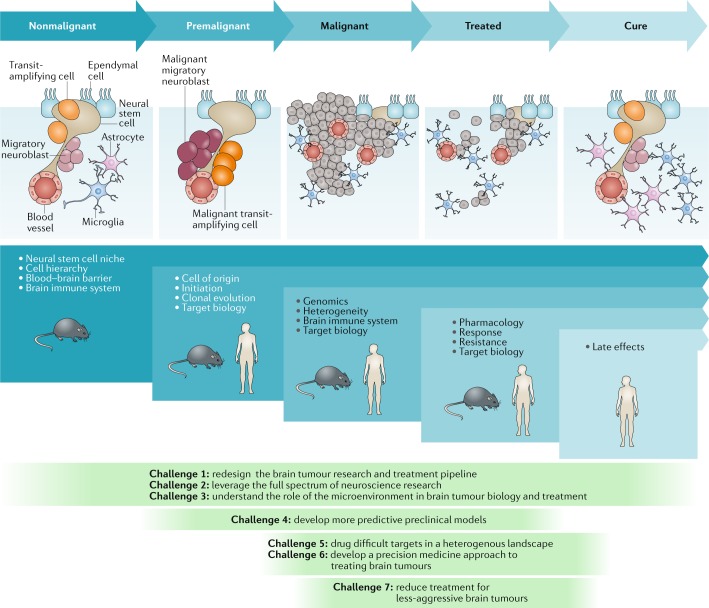
Challenges to curing primary brain tumours at different stages of tumour development. The nonmalignant cellular composition of the ventricular–subventricular zone includes neural stem cells that divide and produce transit-amplifying cells. Transit-amplifying cells give rise to migratory neuroblasts^90^. Ependymal cells are also present in the neural stem cell niche. The niche is intimately associated with blood vessels and might also communicate with other cell types, including microglia and astrocytes. Malignant transformation of neural stem cells presumably leads to the premalignant expansion of transit-amplifying cells and migratory neuroblasts as the nonmalignant hierarchy begins to transform. This unregulated hierarchy generates the malignant brain tumour. As these lesions are treated, tumour cells are killed, with the ultimate goal of eventual cure. The blue panels below these cartoons denote the focus of key research questions at each stage, from nonmalignant brain tissue to the development of cancer and remission following successful treatment. The green panels depict how the seven challenges to progress relate to these specific stages of disease development.

To begin to address these issues and improve the outcomes of patients with brain tumours, Cancer Research UK (CRUK) convened an international panel of brain cancer researchers with interests in neurobiology, preclinical tumour modelling, genomics, pharmacology, drug discovery and/or development, neuropathology, neurosurgery, imaging, radiotherapy and medical oncology, with the task of identifying the most important challenges that must be overcome if we are to eventually be in the position to cure all patients with a brain tumour. In this Position Paper, we summarize seven key challenges identified by the panel that should serve as the foci for future research and investment (Fig. 1). Each of these challenges is worthy of extensive discussion and review, which are beyond the scope of this manuscript. Therefore, we highlight these challenges as a ‘call-to-arms’, summarizing the nature and importance of each challenge rather than providing an exhaustive review of the current level of understanding.

## Challenge 1: redesign research pipeline

Clinical trials have yet to reveal an effective therapy for most brain tumours. This harsh reality stems, in part, from an incomplete understanding of brain tumour biology and the existence of a disconnect between preclinical drug development and rigorous testing in the clinic. Each element of the brain tumour research pipeline, from basic neurobiology to clinical trials, requires careful scrutiny and increased investment, although the development of an overarching strategy that facilitates and promotes interdisciplinary research is equally important (Fig. 1). This strategy would bring an end to the previous ‘siloed’ organization of working practices, in which basic and clinical researchers performed their studies independently and collaborated only when laboratory research was judged to be ready for the clinic or when the laboratory is engaged to understand the reasons why a promising drug failed to achieve the expected level of efficacy in clinical trials. Much deeper, longitudinal collaboration is essential in order to drive progress as rapidly as possible.

Nascent attempts to unify the brain tumour research pipeline are underway. The international paediatric brain tumour community has demonstrated remarkable levels of collaborative activity over many years and is now working to discover and define the genomic subtypes of paediatric brain tumours and form multidisciplinary teams in order to design preclinical studies that better inform the design of clinical trials^11,12^. However, the community must go further, by forming international collaborations that extend beyond the borders of traditional research disciplines and by engaging the entire breadth of expertise available within the biological and physical sciences (see Challenge 2). Such congregations of experts could provide a more comprehensive understanding of the workings of the brain and how these processes are subverted during malignant transformation.

**Fig. 2:**
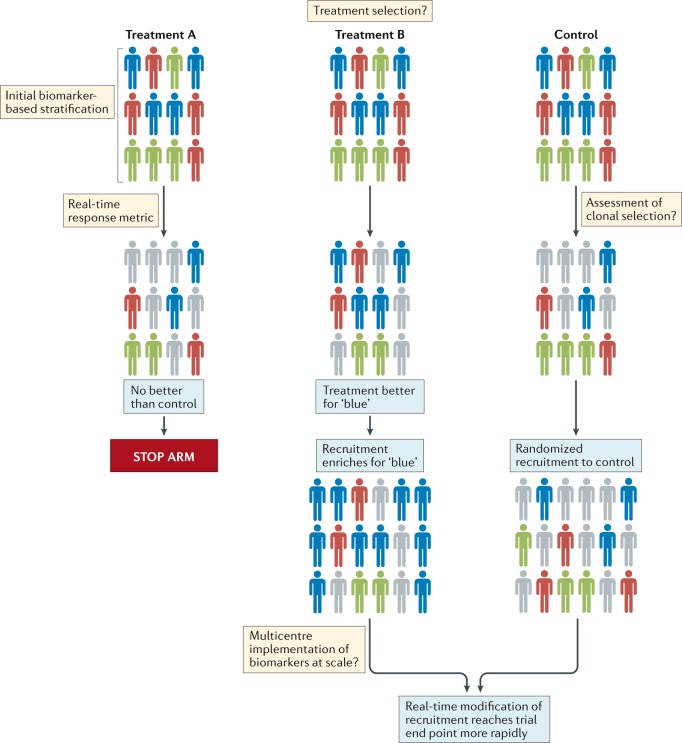
Suggested general outline of a platform approach to designing clinical trials for the assessment of brain tumour therapies. Patients with different molecular subtypes of a specific type of brain tumour (represented as blue, red and green figures) are enrolled into the trial. Patients in the control group receive the optimal standard of care. New treatments can then be tested as comparators in groups A and B. Following a defined period of treatment, the responses of patients in these groups are compared with those of patients in the control group. Responding patients are represented by bold colours; nonresponding patients are coloured grey. In this example, new treatment B produced a biased favourable response in patients with a single disease subtype (blue figures) relative to the control treatment. No advantage of new treatment A is observed relative to the control treatment. Therefore, in the next phase of the study, the new treatment A group is discontinued, while the new treatment B arm proceeds, with enrichment for patients with tumours of the ‘blue’ subtype. The control group continues with randomized recruitment of patients with all disease subtypes. The success of this approach is dependent upon addressing the key challenges summarized in the thought bubbles emerging at each stage of the trial. Many of these challenges are yet to be addressed in patients with brain tumours.

The community should also explore innovative methods of designing and delivering clinical trials. Interest among the brain tumour research community in the use of adaptive trial designs is particularly welcomed^13,14^. Such trials could provide prospective opportunities to modify ongoing studies and integrate the investigation of new hypotheses as data are accumulated and analysed (Fig. 2). This approach has distinct advantages over the less flexible, traditional trial designs, which are typically designed to test one or two primary hypotheses, typically in heavily pretreated patients over a number of years. However, several hurdles must be overcome before novel trial designs can be implemented successfully, including identifying a cadre of rational therapies to feed into these new trials; developing robust biomarker-based patient selection criteria; constructing systems that enable the provision of real-time, in-trial, biomarker and end point data; and formulating optimal mechanisms of generating and sharing complex research data among centres worldwide (Fig. 2). Notwithstanding these challenges, improving the current approach to clinical trials is an urgent objective for the community. In the context of small, genomically defined patient subgroups, international collaborative clinical trials are crucial. A comprehensive review of the current logistical, regulatory and financial hurdles that inhibit the initiation of such trials is therefore warranted.

Greater integration of research disciplines will also require a change in the current research culture. The principal metrics used to reward success in most academic environments are focused on competitive activities, such as publication in high-impact journals and the acquisition of grant funding. These metrics have some value in enabling the assessment and quantification of the extent of innovative thought and discovery, although they arguably also work against the development of the deeper levels of collaborative activity that will ultimately be required to cure patients with brain tumours. This change in academic culture must go beyond a cursory recognition of the value of ‘team science’. Rather, akin to the complex and multidisciplinary groups that operate in the pharmaceutical industry, new reward and promotion structures should be developed for laboratory and clinical researchers alike that inspire and encourage their participation in collaborative academic brain tumour research.

## Challenge 2: use neuroscience research

We now have a considerable level of understanding of the cellular hierarchies and signals that generate and maintain brain activity in the absence of cancer^15^. Specialized glial cells serve as neural stem cells (NSCs) in both the embryonic and adult brain, in which they reside in neurogenic niches and produce daughter cells that differentiate into neurons, astrocytes and oligodendrocytes (Fig. 1). These niches also include supporting cells and secreted factors that are critical for the close regulation of both NSC self-renewal and the early stages of daughter cell differentiation: an intimate relationship between NSCs and blood vessels seems to be especially important in the neurogenic niche^16^.

**Fig. 3:**
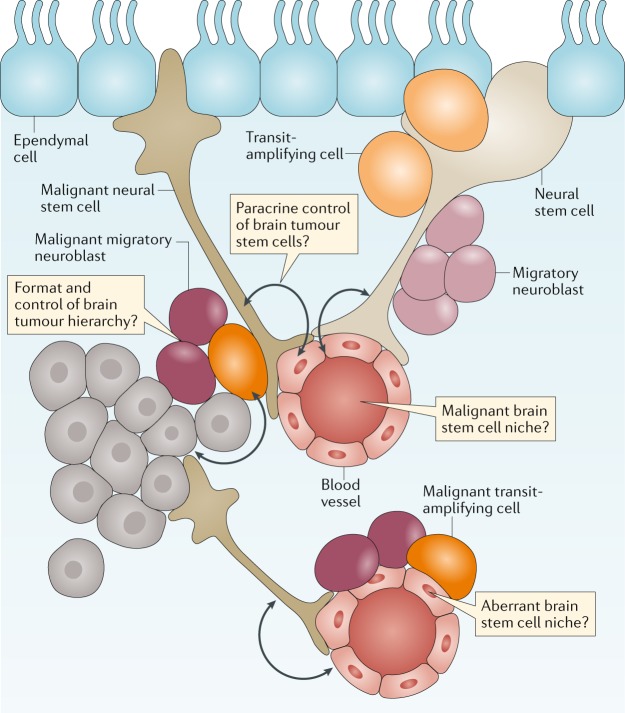
The cellular origins of brain tumours. Brain tumours are thought to arise from a transformed and malignant brain tumour hierarchy generated from transformed neuronal stem cells. Nonmalignant and malignant stem cells in the brain are believed to reside in perivascular niches formed by the blood vessels of the brain. Unaddressed key research questions that could provide a better understanding of brain tumour development and treatment are shown in the thought bubbles.

Several lines of evidence suggest that brain tumours arise within, or are driven by, cells that recapitulate the neurogenic niche (Fig. 3). Stem-like cells have been isolated from paediatric and adult brain tumours^17–19^, and brain tumours have been shown to contain malignant niches that seem to recapitulate the micro-architectural features and signalling properties of the nonmalignant neurogenic niche^20,21^. Recurrent mutations in brain tumours can also perturb the signalling pathways that regulate brain development^12^, and subgroups of brain tumours have been shown to contain the transcriptomes and epigenomes of their originating parental NSCs that generated these tumours in the mouse brain^22–29^.

Evidence that brain tumours are a consequence of aberrant brain development or repair underscores the importance of closer integration between neurobiological and cancer research; however, clear examples of such collaborations are rare. Only ~1% (US$260 million) of the $21 billion invested in neuroscience research by the US NIH in 2018 is aimed specifically at brain tumour research. Furthermore, although the PubMed database currently contains ~320,000 and 198,000 articles on neuroscience and brain tumour research, respectively, only 6,000 (~2% and ~3.5%, respectively) overlap. These observations are crude indicators of the level of interaction, but they suggest that the brain tumour research community is not capitalizing fully on the available research data. An increased focus on areas of common interest, such as immune dysregulation in patients with cancer and in those with dementia, should drive joint funding initiatives and ultimately result in progress for patients^30,31^.

## Challenge 3: understand the TME

**Fig. 4:**
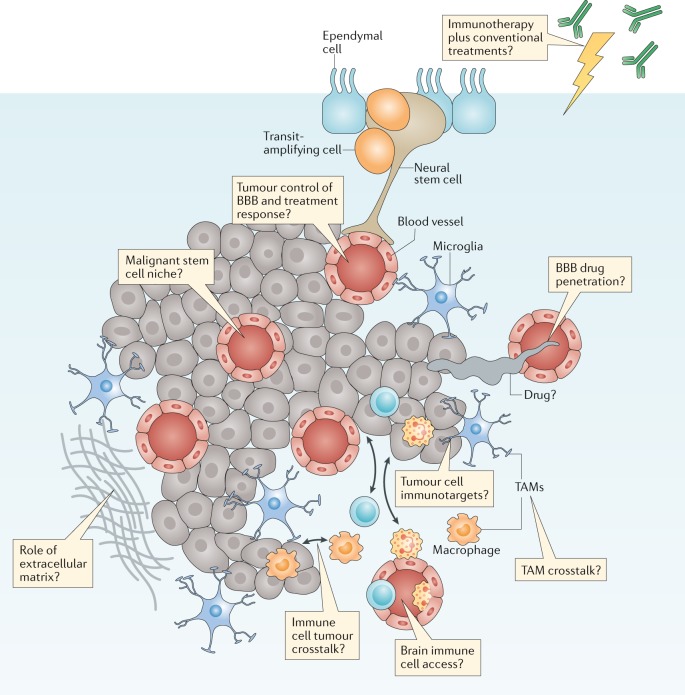
The brain tumour microenvironment and treatment. Brain tumours typically comprise a complex mixture of malignant cells and a great variety of nonmalignant cells. These include immune, vascular and nonmalignant brain cells, all of which are able to communicate with malignant cells and contribute to the development and progression of cancer. These features also form a key part of the response of a tumour to treatment. Key research questions focused on understanding how these elements affect tumour biology and responses to treatment are shown in the thought bubbles. BBB, blood–brain barrier; TAM, tumour-associated macrophage.

A thorough understanding of the properties and functions of the tumour microenvironment (TME) is required in order to obtain a complete understanding of brain tumour biology and treatment^9,32^ (Fig. 4). As discussed with regard to Challenge 2, closer interactions with scientists investigating nonmalignant brain diseases will be especially important to this effort. Over many decades, research groups studying neurodegeneration and other nonmalignant brain diseases have generated a considerable level of understanding of the biology of the extracellular matrix, of specific brain-resident cellular populations (such as microglia, astrocytes and neurons) and of blood vessels (including those forming the BBB) that comprise the TME.

The immune and vascular components of the TME are likely to have particular relevance for improving the treatment of brain tumours^6,33^. For example, the discovery of the immune or glymphatic system of the brain and evidence that immunotherapies are effective treatments for an increasing number of cancers have prompted studies of the efficacy of such therapies in patients with brain tumours^31,34–36^. The major myeloid cell populations of the brain TME comprise macrophages and microglia — together referred to as tumour-associated macrophages (TAMs)^37,38^. Glioblastoma cells stimulate TAMs to produce immunosuppressive, tumour-promoting cytokines and enhanced apoptosis of T cells^39^. Glioblastoma cells can also inhibit the production of immunostimulatory cytokines and activate the recruitment of regulatory T cells, thus inhibiting antitumour immune responses^40^. These data suggest that inhibiting the activity of TAMs might provide therapeutic benefit in patients with brain tumours, although the clinical reality is likely to be more complex. Research conducted over the past decade suggests that re-educating TAMs to adopt phenotypes that are likely to prevent or inhibit the progression of brain tumours might be more effective than depleting all TAM populations^32,41^. A range of additional immune-based therapies are also currently in development, including vaccines, cellular therapies (such as chimeric antigen receptor (CAR) T cells) and immune-checkpoint inhibitors^9,31,34,40–42^.

Regardless of the type of immunotherapy that is being developed, several key challenges must be addressed if we are to deploy these agents effectively in the treatment of brain tumours (Fig. 4). Once again, a better understanding of tumour biology will be important to the selection of patients who are most likely to benefit from therapies and to improve the level of understanding of treatment-related toxicities (see Challenge 1). Addressing these challenges will not be straightforward for immunotherapies, not least because even the imprecise predictors of a response to immune-checkpoint inhibition in other cancers, such as tumour mutational burden and/or DNA methylation, remain to be proved in patients with brain tumours^43^. Furthermore, the identity of the optimal targets of other immunotherapies, such as CAR T cells, which might be patient specific, also remains unclear. Other important and unanswered questions surrounding the use of immunotherapies in patients with brain tumours include the degree to which the BBB will impede the access of cellular and molecular immunotherapies to tumours; whether immunotherapies will synergize with or counteract the effects of existing treatments, such as temozolomide, steroids and/or radiotherapy; and the type and severity of neurological adverse effects associated with immunotherapies and how best to prevent, monitor and manage these. Thus, although immunotherapy holds great promise as a new therapeutic approach for the treatment of patients with brain tumours, many aspects of the unique immunological and microenvironmental status of the brain present challenges that need to be overcome in order to develop and validate these therapies efficiently^9^.

As previously alluded to, the BBB is a major hurdle to the successful treatment of brain tumours^44^. This dynamic structure of tight junctions and molecular pumps comprises endothelial, ependymal and tanycytic cells that communicate with other brain cells and with circulating immune cells^45–48^. Single-cell transcriptomics has revealed a remarkable level of regional heterogeneity along the arteriovenous axis of the BBB, including a seamless continuum of endothelial cells versus a punctuated continuum of mural cells^49^. These insights could prove important in determining the functionality of the BBB in tumours located in different regions of the brain.

Discrete subtypes of brain tumours might also alter the BBB (Fig. 4). In this regard, the WNT subtype of medulloblastomas, which are highly sensitive to treatment, has been shown to secrete WNT antagonists that ‘re-educate’ the BBB, thus causing it to adopt a phenotype that is highly permeable to systemic chemotherapy^6^. Similarly, the therapeutic response of gliomas to temozolomide might be improved by use of this agent in combination with small-molecule inhibitors of WNT signalling^33^. Greater research efforts should be invested in cataloguing the integrity and other alterations of the BBB in each brain tumour subtype. This information might enable the more widespread use of systemic therapies that are currently regarded as BBB non-penetrating in those tumours that lack an intact BBB and support the use of WNT inhibitors and other approaches to enhance the delivery of chemotherapy to those tumours in which the BBB is intact^50^.

## Challenge 4: develop preclinical models

The limited progress made in the treatment of brain tumours relates, in part, to the inaccuracy of preclinical models that have thus far failed to consistently show responses to agents with therapeutic activity in patients. Preclinical drug development pipelines that enable the accurate prediction of effective drugs are especially important for rare cancers, including brain tumours, owing to the low numbers of patients available for participation in clinical research^12,51^. Current pipelines are limited in their ability to identify new, more effective treatments of brain tumours for several reasons: they typically involve poorly characterized in vitro systems or subcutaneous tumour xenografts, rather than more accurate orthotopic models of brain tumours; they do not enable the assessment of benefit from new treatments in terms of survival relative to that provided by the existing combinations of neurosurgery, radiotherapy and/or chemotherapy; and finally, they usually lack rigorous characterization of other clinically important features, such as those of the BBB. Thus, curing all patients with a brain tumour will require a new approach that leverages an improved understanding of neuroscience and brain tumour biology to develop and deploy more accurate preclinical models.

A better understanding of the processes that drive the development and progression of brain tumours should promote the development of more accurate genetically engineered mouse models (GEMMs) and patient-derived xenografts (PDXs). We recommend that the brain tumour community maintains a centralized catalogue of all existing and newly developed preclinical models that have been subjected to a minimum agreed set of rigorous histological, genomic and imaging evaluations. Models from this catalogue should be made readily available and would provide the community with universally accessible, validated models that closely mimic the characteristics of human tumours. Initiatives such as the ITCC Paediatric Preclinical Proof of Concept Platform are already underway, with the intention of creating such a resource.

**Fig. 5:**
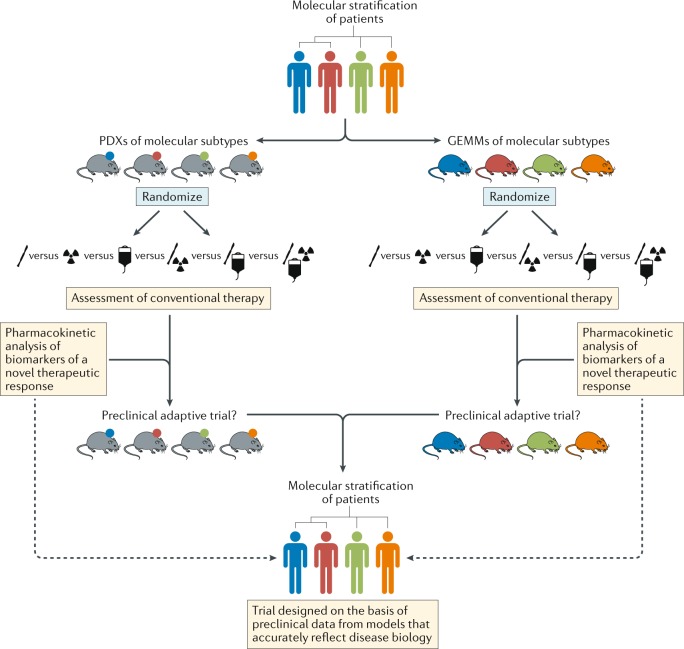
Stratified approaches to the development of new treatments for patients with brain tumours. Schema depicting pathways for improvements in the preclinical development of new treatments for subsets of patients with brain tumours of distinct molecular and/or clinical subtypes (represented by different colours). This approach should include both patient-derived xenografts (PDXs) and genetically engineered mouse models (GEMMs) that accurately reflect the principle molecular and/or clinical subtypes. In order to provide the optimal level of clinical relevance, these models must also involve an assessment of responses to conventional combination therapies. These existing treatments could be included in adaptive preclinical trials designed to test novel treatments and inform the ultimate clinical trial design.

In addition to minimum standards of model characterization, the community should also adopt minimum standards for preclinical studies, thus enabling more effective comparisons of the results of different studies (Fig. 5). Of particular note, although patients with brain tumours receive complex, multimodality therapy, mice in preclinical studies typically receive monotherapies that are rarely comparable to the standard of care for the malignancy that is being modelled. Thus, efforts to establish preclinical ‘mouse hospitals’, in which potential new therapies are tested in the context of combination with neurosurgery, fractionated radiotherapy and/or conventional chemotherapy, should become the new standard^11^. Researchers with access to such preclinical hospitals should also perform robust pharmacokinetic (with particular attention to BBB penetration), pharmacodynamic and biomarker (tumour, liquid biopsy and imaging) studies for novel agents, thus maximizing the speed and likelihood of success of clinical translation (Fig. 5).

The failure of many drugs to penetrate the BBB to any clinically meaningful extent is an important reason for the poor treatment responses of patients with brain tumours to systemic therapies^52^. Functional imaging studies in particular can provide evidence of early treatment responses, although a failure to detect a response cannot distinguish between a drug that is ineffective and one that simply does not cross the BBB. Contrast enhancement of tumours on MRI, using various agents, can reveal disruption of the BBB, for example, following ultrasonography-mediated opening of the BBB^53^; however, this approach does not provide direct evidence of drug delivery. More direct evidence can be provided by observing tumour accumulation on PET imaging of drugs labelled with a positron-emitting isotope, such as [methyl-^11^C] temozolomide in patients with gliomas^54^. However, conjugating all new drugs with such isotopes — particularly with ^11^C, which will not perturb drug chemistry and thus drug–target interactions but has a sufficiently short half-life (20.3 minutes) — would be a formidable undertaking. Thus, the community should agree on acceptable preclinical and clinical standards for demonstrating successful drug delivery to brain tumours.

In addition to being grown as PDXs, patient-derived brain tumour cells can be consistently and successfully encouraged to propagate under NSC culture conditions as organoids^55,56^. These short-term culture systems have enormous potential for studying interactions between different cells and drug sensitivities and for interrogation using genome-wide interference and/or editing techniques^20,55–58^. Preclinical pipelines that deploy these novel screening approaches, together with the use of matched GEMMs and PDX models (ideally in humanized mice), should provide a more comprehensive and more accurate means of delivering optimized drugs and their associated biomarkers into clinical trials^11^.

Preclinical and clinical research efforts to evaluate the value of new treatments, relative to that of the conventional treatments of brain tumours, should also consider re-evaluating some long-established treatment approaches that might be of questionable value. For example, corticosteroids, such as dexamethasone, continue to be used perioperatively and might be continued throughout subsequent treatment. The original reasons for using dexamethasone in this way probably reflect antiproliferative activity that confers protection of nonmalignant cells from radiotherapy-induced and chemotherapy-induced genotoxic stress. However, both clinical data and preclinical data from mouse models suggest that corticosteroids might decrease the effectiveness of treatments and shorten the survival durations of patients with glioblastoma. Thus, greater efforts should be invested in identifying alternative agents, on the basis of robust preclinical and clinical evidence, such as anti-VEGF agents for the treatment of brain tumour-associated oedema^59^.

## Challenge 5: drugging complex cancers

The so-called omics revolution is providing a comprehensive view of the genomes, epigenomes and transcriptomes of brain tumours; this additional information has enabled the segregation of histologically similar subsets of tumours into clinically and molecularly distinct subgroups. However, despite a few notable exceptions, such as inhibitors of mTOR or BRAF in selected patients^60,61^, genomics has yet to deliver on the promise of identifying new therapeutic targets in patients with brain tumours. Discovering new treatments of brain tumours will require a much deeper level of understanding of the biology of potential drug targets, combined with the application of cutting-edge drug discovery approaches to inhibit these targets.

Progress in drug development will not come easily. Alterations identified in brain tumours in the past decade include poorly understood targets that are frequently regarded as undruggable, including amplification of genes encoding transcription factors, such as *MYC* and *MYCN*; gene translocations, such as *FGFR–TACC* and *C11OF95–RELA*^27,62^; oncogene ‘enhancer hijacking’, for example, by *GFI1* (ref.^63^); and mutated histones, such as *H3F3A* and *HIST1H3B* variants^64,65^. Furthermore, these alterations exist in a shifting landscape of tumour heterogeneity, in which the populations of clones carrying each alteration fluctuate as the disease evolves in response to therapy^66,67^. This clonal evolution means that the proportion of tumour cells carrying a specific drug target can vary markedly during the course of disease, thus complicating efforts to target specific alterations. This heterogeneity can be measured in terms of mutational load, epigenetic alterations, rewiring of transcriptional circuits and microenvironmental influences and is widely considered to underpin the diversity of responses to treatment. The serial analysis of tumour biology using additional measures, such as advanced metabolic imaging and analyses involving serial sampling of circulating tumour DNA, is discussed with regard to Challenge 6 and might provide a more complete and accurate picture of tumour evolution.

Although daunting, the data provided by these novel approaches reflect an important principle that must be adopted by brain tumour researchers and clinicians — the notion that brain tumours are monogenetic and monoclonal must be dispelled. Comprehensive mapping of genomic evolution before, during and following treatment, including the use of single-cell sequencing approaches, should be used to better identify and prioritize treatment targets^68,69^. This objective will be best achieved in the context of prospective clinical trials, in which molecular imaging and other diagnostic approaches can be used to provide real-time assessments of disease evolution^70^. Prioritized targets should also be interrogated using approaches, such as deep mutational scanning, that can reveal information on the intrinsic properties of proteins and their function and how this changes with mutation^71^. Reverse translation efforts involving detailed mechanistic studies after a clinical trial of appropriately prioritized targets should include functional studies that enable the accurate definition of target function in tumours. Such serial analyses will likely be complex and resource-intensive and are probably best organized as part of a community-wide approach. This approach might be promoted in several ways: development of international tumour subtype-specific trials; the widespread availability of brain tumour stem cell banks (such as the UK glioma cellular genetics resource (GCGR), the human glioblastoma cell culture (HGCC) resource in Sweden and the Stand Up to Cancer (SU2C) Canada Cancer Stem Cell Dream Team); established efforts to understand complex cancer targets (such as the US National Cancer Institute RAS Initiative); and closer collaborations with industry.

## Challenge 6: precision medicine

The current classifications of brain tumours are based primarily on microscopic morphology and immunohistochemistry^72^. This approach enables the broad characterization of tumour type and a certain level of prognostication, although it fails to capitalize on the wealth of clinically relevant insights produced by the genomic subtyping of brain tumours. This dilemma is exemplified by medulloblastoma and ependymoma, both of which consist of clinically relevant subtypes with discrete cells of origin, driver mutations and global transcriptomic and epigenomic patterns that are yet to be incorporated into the WHO classification of central nervous system tumours^10,12,72–75^.

Concerns that genome-wide classification tools might prove impractical for the routine diagnosis of brain tumours have been dispelled by the successful large-scale methylome subtyping of formalin-fixed, paraffin-embedded tumours^75,76^. Therefore, the challenge to the brain tumour community is not whether but how to deploy diagnostic genomic profiling. In the UK, the centralization of genomic profiling through the National Health Service provides an enormous opportunity to begin this process at a population level. In doing so, it will be important to note that not all brain tumours are created equally: paediatric medulloblastomas and ependymomas comprise robust subgroups with distinct characteristics. However, the degree of diagnostic separation of other brain tumours, including gliomas in adults, is less clear.

The genomic classification of brain tumours will increase the level of diagnostic precision that is currently achievable, although intratumoural heterogeneity and the often limited amounts of tumour material available for analysis are likely to confound the accurate diagnosis of some tumours^68,77^. Therefore, the research community should aim to urgently investigate the use of more advanced imaging techniques, such as ^13^C-hyperpolarized MRI, radiomics and sequencing of cell-free DNA obtained from plasma and/or cerebrospinal fluid, as ancillary approaches to the diagnosis and classification of brain tumours^70,78–80^. The challenge of validating and combining these new diagnostic modalities is enormous but also holds the potential to greatly improve the accuracy of brain tumour classification. Implementation of such approaches might also improve the assessment of novel therapies as part of the federal drug approval process. In this regard, the current standards, such as the Response Assessment in Neuro-Oncology criteria^81^, which describe complete and partial drug responses, are of limited value for assessing new treatments of slow-growing tumours, such as low-grade glioma. Early molecular and/or metabolic responses that are predictive of ultimate clinical benefit might, therefore, provide a more accurate means of evaluating the efficacy of treatments.

Once validated, new classification tools, such as those outlined above, must be adopted within the WHO guidelines in order to enable use by the wider community. A comprehensive strategy to achieve this goal should formally include genomic metrics, such as transcriptomic analysis, in WHO diagnosis and classification schemes; involve appropriate training of experienced and newly qualified pathologists in genomic methodologies; be accompanied by appropriate levels of investment in the resources (both capital and personnel) needed to deliver genomic assays routinely in diagnostic pathology labs; and incorporate standardized genomic characterization within both conventional and clinical trial management strategies. Histology will always have a place in the diagnosis of patients, although only when used in concert with advanced molecular and imaging-based diagnostics will this provide a comprehensive prediction of tumour behaviour.

**Fig. 6:**
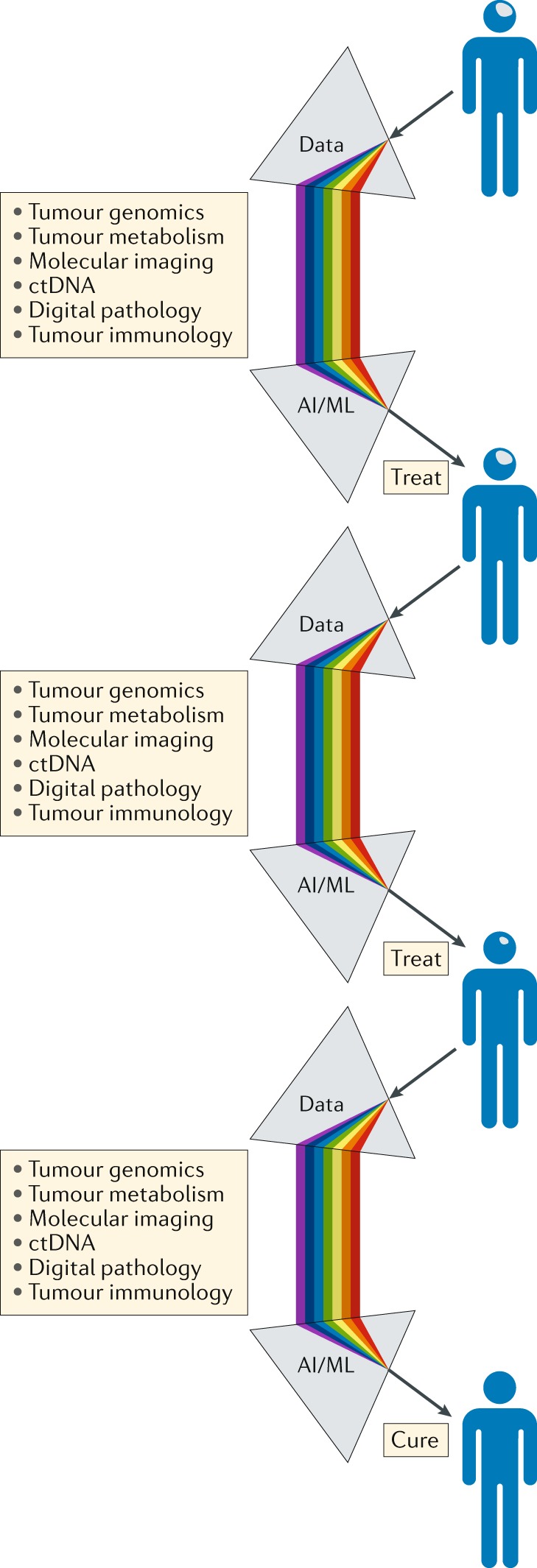
A precision medicine approach to the treatment and management of patients with brain tumours. Serving as a ‘biological prism’, the use of artificial intelligence (AI) and machine learning (ML) approaches should enable the integration of separate data streams at critical points in the patient journey, providing an unprecedented level of comprehensive decision-making to guide the selection of the most appropriate treatment and support the management of patients with brain tumours. The knowledge gained through the management of each patient should be analysed iteratively over time, enabling constant refinement and improvements in clinical decision-making. ctDNA, circulating tumour DNA.

In addition to developing advanced diagnostic approaches, we must also learn how best to integrate these rich data streams if they are to revolutionize the treatment of patients with cancer (Fig. 5). Continual, iterative and integrated analysis of these complementary and complex data streams should be made possible by machine learning, artificial intelligence and/or other mathematical and computational approaches. This will require a concerted effort to invent and deploy new analytical approaches and visualization technologies in order to generate a single flow of information that travels with a patient throughout his or her treatment journey, enabling better-informed management decisions and guiding the patient towards the maximum opportunity for cure (Fig. 6). Reporting the clinically relevant findings from such large-scale data sets to health-care professionals and patients will require a multidisciplinary approach, supported by appropriate information technology infrastructure, genetic counsellors, medical geneticists and clinical scientists. This longitudinal and integrated approach will also enable the development of the next generation of adaptive precision medicine clinical trials.

## Challenge 7: reduce treatment for some

Certain brain tumours, particularly those arising in children, can be cured with aggressive surgery, radiotherapy and chemotherapy, although these cures often carry a considerable cost, particularly for young children who have lifelong neurocognitive and endocrine adverse effects^4,82^. Reducing the intensity of treatment in children with other cancers, such as acute lymphoblastic leukaemia, has enabled a reduction in the incidence of adverse effects while maintaining high cure rates; however, this approach has proved challenging to implement in patients with brain tumours, the majority of whom have disease progression following surgery alone. Identifying which patients might be cured with less-intense adjuvant radiotherapy and chemotherapy and to what extent the intensity of these modalities can be safely reduced remains a major challenge.

Medulloblastomas provide a clear example of how a detailed understanding of tumour biology can guide adjustments of treatment intensity. Historical attempts to limit the damaging effects of craniospinal radiotherapy on the developing brain by reducing the radiation dose have failed, largely owing to an inadequate understanding of medulloblastoma biology, which precluded the accurate selection of patients with truly low-risk disease^83–85^. A breakthrough was achieved with the recognition that almost all patients with the WNT subtype of medulloblastoma are cured^12^. This discovery has led to a series of highly selective ongoing studies testing reduced-intensity radiotherapy in patients with this disease subtype (NCT02724579, NCT02066220, NCT02212574 and NCT01878617). Evidence that WNT-subtype tumours (but not other forms of medulloblastoma) lack a BBB and are therefore remarkably vulnerable to systemic chemotherapy provides an explanation for the curability of these tumours, thus opening further options for alternatives to radiation-based treatments^6^. Paediatric low-grade gliomas provide another example of how molecularly guided treatments (such as inhibitors of activated BRAF and MEK) are enabling reductions in radiation dose^86,87^. Studies involving patients with other brain tumours are likely to identify similar approaches for the rational de-escalation of therapy.

A comprehensive approach to preventing and ameliorating the adverse effects associated with long-term treatment should include prospective functional and patient-reported outcome measures, as well as innovative predictive assessments of risk. Novel ways of supplementing these data are also emerging in the form of genetic polymorphisms and somatic genomic characteristics that might predict the risk of subsequent intellectual disability and/or hearing loss^88,89^.

## Conclusions

The past 15 years have witnessed a revolution in our understanding of cancer. The integration of genomic and developmental biology has shown that morphologically similar cancers comprise discrete subtypes, driven by different genetic alterations, which likely arise from distinct cell lineages. These data help to explain why cancers once regarded as histologically homogeneous diseases have a discrepant range of characteristics. Improved understanding is also leading to the development of completely new treatment approaches for cancer, such as immunotherapies, and novel ways to test such therapies, such as adaptive trial designs. However, the successes achieved with these improvements have not occurred equally across all forms of cancer. Of particular note, the treatment of most childhood and adult brain tumours is at an impasse, with no new, more effective therapies being developed in the past 30 years. Thus, all available evidence suggests that the current preclinical and clinical research approaches to curing brain tumours are ineffective.

Herein, we have summarized seven major challenges to progress that must be overcome if we are to produce the sea change in brain tumour therapy that has long been awaited by patients and their families. Some of these challenges might seem to be self-evident; however, each will require a profound change in research and clinical practice and investment. In order to truly redesign the brain tumour research and treatment pipeline and to leverage the full spectrum of neuroscience research, modest improvements in the level of interdisciplinary collaborations among otherwise siloed research groups will not be sufficient. We envisage a much more substantial congregation of experts and infrastructure, focused on the task of curing brain tumours. This call reflects the need to practise brain tumour research differently and to identify, convene and support teams of scientists and clinicians who focus specifically and urgently on the need to improve the lives of patients with brain tumours. This approach must be different from the largely ad hoc and predominantly poorly structured brain tumour collaborations and programmes that currently exist in most academic centres. The harsh reality is that the current efforts at various universities and aligned clinical environments around the world have failed to adequately improve the understanding and treatment of patients with brain tumours. Nevertheless, this approach would probably be easiest to implement within the context of existing academic settings in order to engage with and closely incorporate disciplines not traditionally involved in brain tumour research, such as engineering, chemistry, physics and mathematics.

As we seek to better understand the role of the TME and aim to develop more predictive preclinical model systems, we need to take a long, hard look at the current model systems. Many of these clearly do not adequately reflect all the nonmalignant and malignant cell types that compose brain tumours and have failed to accurately predict treatments that will be effective for testing in clinical trials. Only systems that are proven to model clinically relevant aspects of tumour biology should be used as evidence to guide the initiation of clinical trials. Furthermore, through reverse translation, we should refine the use of or entirely remove models that fail to predict clinical efficacy.

Concerns relating to safety and patient acceptability limit the ability to obtain repeat biopsy samples from brain tumours in most patients, thus preventing longitudinal studies of tumour biology and treatment response and the deployment of a precision medicine approach. The use of liquid biopsies and advanced imaging approaches should be explored immediately to provide additional approaches to determine tumour response earlier in the course of treatment. Finally, the challenge most likely to yield patient benefit in the immediate future will be the use of advanced stratification approaches to reduce the risk of treatment-related toxicities, especially among children.
